# *Enterococcus* and *Streptococcus* spp. associated with chronic and self-medicated urinary tract infections in Vietnam

**DOI:** 10.1186/1471-2334-12-320

**Published:** 2012-11-23

**Authors:** Louise Ladefoged Poulsen, Magne Bisgaard, Nguyen Thai Son, Nguyen Vu Trung, Hoang Manh An, Anders Dalsgaard

**Affiliations:** 1 Department of Veterinary Disease Biology, Faculty of Health and Medical Sciences, University of Copenhagen, Groennegaardsvej 15 DK-1870, Frederiksberg C, Denmark; 2 Hospital 103, Military Medical University, Ha Dong, Hanoi, Vietnam; 3 Department of Medical Microbiology, Hanoi Medical University, Hanoi, Vietnam

## Abstract

**Background:**

Urinary tract infections (UTI) are one of the most common infections among women worldwide. *E. coli* often causes more than 75% of acute uncomplicated UTI, however, little is known about how recurrent UTIs and indiscriminate use of antimicrobials affect the aetiology of UTIs. This study aimed to establish the aetiology of UTI in a population of recurrent and self-medicated patients referred from pharmacies to a hospital in Hanoi, Vietnam and to describe genotypes and antimicrobial susceptibility of the associated bacterial pathogens. The aetiology of bacterial pathogens associated with UTI (defined as ≥ 10^4^ CFU/ml urine) was established by phenotypic and molecular methods. *Enterococcus faecalis* isolates were typed by Multi Locus Sequence Typing (MLST), Pulsed-Field Gel Electrophoresis (PFGE) and antimicrobial susceptibility testing.

**Methods:**

Urine samples from 276 patients suffering symptoms of urinary tract infection were collected and cultured on Flexicult agar® allowing for detection of the most common urine pathogens. Patients were interviewed about underlying diseases, duration of symptoms, earlier episodes of UTI, number of episodes diagnosed by doctors and treatment in relation to UTI. All tentative *E. faecalis* and *E. faecium* isolates were identified to species level by PCR, 16S rRNA and partial sequencing of the gro*EL* gene. *E. faecalis* isolates were further characterized by Multi Locus Sequence Typing and antimicrobial susceptibility testing.

**Results:**

Mean age of 49 patients was 48 yrs (range was 11–86 yrs) and included 94% women. On average, patients reported to have suffered from UTI for 348 days (range 3 days-10 years, and experienced 2.7 UTIs during the previous year). Cephalosporins were reported the second drug of choice in treatment of UTI at the hospital. *E. faecalis* (55.1%), *E. coli* (12.2%) and *Streptococcus gallolyticus subsp. pasteurianus* (8.2%) were main bacterial pathogens. MIC testing of *E. faecalis* showed susceptibility to ampicillin, penicillin and vancomycin, but high-level resistance against gentamicin (48.1%). MLST revealed 12 Sequence Types (ST) of which ST 16 made up 44.5% and showed closely related PFGE types.

**Conclusion:**

The different aetiology of UTI compared with reports elsewhere, where *E. coli* dominates, may be a result of chronic and recurrent UTIs together with indiscriminate use of antimicrobials. The similar genotypes shown by epidemiologically unrelated ST 16 isolates in Vietnam and elsewhere, suggest that *E. faecalis* ST 16 might represent a globally distributed clone. Treatment of UTI with cephalosporins may select for *E. faecalis* as it is intrinsic resistant and further studies are needed to establish the source(s) and role of *E. faecalis* ST 16 in acute UTI.

## Background

Urinary tract infection (UTI) represents one of the most common bacterial infections and it is estimated that 50% of all women will experience at least one episode of UTI during their life time [1,2]. The most frequently isolated pathogen is *E. coli* making up 50% to 70% of UTI cases [3]. Common laboratory diagnostic test for UTI include; leucocyte esterase test, nitrite test and culture for bacterial pathogens [4,5]. Although enterococci seem associated with a relative low percentage of community-acquired UTI’s [6], the prevalence of enterococci have increased in hospital-acquired infections in general, and now represent a common cause of hospital-acquired infections after *E. coli* and *S. aureus*[7]. Enterococci are intrinsic resistant to a number of antimicrobials [8] and can further easily acquire resistances which increase their potential to cause disease and spread in hospital environments [8]. The acquisition of high-level gentamicin resistance by enterococci in particular represents a health problem, since gentamicin is used in combination with cell-wall active compounds, i.e. ampicillin, penicillin or vancomycin, for treatment of endocarditis associated with enterococci [9].

Little is known about the incidence and aetiology of UTI in Vietnam and other less developed countries. This is partly because UTIs are most often not reportable diseases and self medication is common, so many infections are treated by patients or pharmacists alone [10]. It has previously been described that Vietnamese women use various forms of self-treatment to prevent and cure reproductive tract infections which may be potentially harmful and may favor opportunistic infections [11].

The objective of this study was to investigate the aetiology and patient characteristics of UTI among patients seen at a hospital in Hanoi, Vietnam, and establish the genotypes and antimicrobial resistance of *E. faecalis,* which appeared as the most common associated pathogen.

## Methods

### Recruitment of patients and urine collection

All urine samples were collected from January 2008 to January 2010 at the Military Medical University, Hospital 103 in Ha Dong, Hanoi. Nearby pharmacies were instructed to refer patients to Hospital 103 that reported at least one of the following clinical symptoms of UTI: frequent urination, painful urination, hematuria, cloudy urine, pain in pelvic area or lower back. A midstream urine sample was collected at the hospital under supervision of a nurse and bacteriological culture initiated immediately after collection. Based on the established aetiology and results of antimicrobial susceptibility testing, patients were informed about which antimicrobial agent should be used for treatment. All patients were informed orally and in writing in their own mother tongue about the possibility to participate in the study and withdraw at any time without explanation. After the information was given, patients signed an approved consent form. The study protocols were approved by the ethical committee at the Hospital 103. No patients refused to participate.

### Bacterial culture of urine

Flexicult agar plates (Statens Serum Institute, Copenhagen, Denmark) were used for culturing urine [12]. According to the manufacturer pure cultures with ≥ 10^3^ CFU per ml urine should be regarded positive, however, as a conservative measure and due to the findings of unusual aetiology we used a definition of ≥ 10^4^ CFU per ml urine as a positive urine sample. The selective and indicative Flexicult agar plate allows for the detection of the 10 most common pathogens associated with UTI, including *E. coli, Klebsiella* spp*., Enterobacter* spp*.,* and *Proteus* spp*.*[12]. Urine samples from a total of 276 patients were poured over Flexicult plates immediately after collection and incubated at 37°C for 18–24 hrs and if plates showed no visible growth for another 18–24 hrs. If plates showed growth of *E. coli, E. faecium* or *E. faecalis* in pure culture with ≥ 10^4^ CFU per ml urine, three individual colonies were randomly picked from the control compartment in the plate and sub-cultured on non-selective LB agar, Lennox plates (Difco^TM^, Becton, Dickinson and Company, Sparks, USA) which were incubated overnight at 37°C to obtain pure cultures. Colonies were subsequently grown overnight at 37°C in Brain Heart Infusion broth (Oxoid, Basingstoke, Hampshire, England) and stored for further characterization at – 80°C in Cryo tubes containing 30% glycerol. The identity of the three colonies was determined to confirm pure culture in the urine samples.

### Patient questionnaire

All patients were interviewed when urine samples were taken. Registered personal data included: age and sex as well as self-reported underlying diseases, including hematological disorders, respiratory infections, diarrhoea, diabetes, cancer, HIV/AIDS, liver cirrhosis, alcoholism, anatomical malformations of urinary tract, and history of nephro- or urolithiasis. Patients reporting underlying diseases as described above as well as hospital-acquired infections were excluded. Thus, study subjects included those with acute, recurrent and/or self-medicated UTI. The following clinical symptoms were recorded: frequent urination, painful urination, cloudy urine, blood in urine, pain in pelvic area, pain in lower back and fever. In addition, information was recorded about duration of symptoms, previous episodes of UTI, number of UTI episodes during the last year and how many of these episodes were diagnosed by a doctor. Finally, medical treatment of UTI before enrolment in the study was registered, e.g. type of antimicrobial used.

### PCR and 16S rRNA identification of enterococci

*E. faecalis* and *E. faecium* were identified by species-specific PCR [13]. Isolates tentatively identified as *E. faecalis* or *E. faecium* based on appearance on the Flexicult agar plate, but testing negative in the *E. faecalis-* /*E. faecium-*specific PCR (n=9), were characterized by 16S rRNA sequence analysis (Macrogen®, Seoul, South Korea) [14] using primers modified after [15]. Species identification was done by BLAST search in GenBank by use of the EzTaxon server ( http://www.eztaxon.org/).

### Partial sequencing of *groEL*

The identity of *Strepcococcus gallolyticus* subsp. *pasteurianus* as shown by 16S rRNA sequence analysis was confirmed by partial sequencing (Macrogen®) of the *groEL* gene [16] which encodes a heat-shock protein. Sequences were assembled using CLC Main Workbench 5.2 software (CLC bio, Aarhus, Denmark) and species identification was done by blasting the sequences in the NCBI database ( http://www.ncbi.nlm.nih.gov/). To determine a more precise similarity between the obtained sequences and the sequence of the type strain, local pair wise sequence alignment was done, using EMBOSS water alignment ( http://www.ebi.ac.uk/).

### Antimicrobial susceptibility testing

MIC were determined for 27 *E. faecalis* to ampicillin (AMP, 2–32 μg/ml), penicillin (PEN, 2–32 μg/ml), vancomycin (VAN, 1–32 μg/ml) and high-level gentamicin (GEN, 16–1024 μg/ml) using the Sensititre® system (Trek Diagnostics Systems, East Grindstead, England). The following breakpoint values as proposed by EUCAST (European Committee on Antimicrobial Susceptibility Testing; http://www.eucast.org/) were used: AMP R > 8 μg/ml, VAN R > 4 μg/ml where as R > 128 μg/ml was used for high-level GEN resistance.

### Identification of *aac (6’)-Ie aph (2”)-Ia* resistance gene

All strains which had a MIC ≥ 512 μg/ml and three control strains which had a MIC ≤ 16 μg/ml against gentamicin were tested by PCR for the presence of the *aac (6’)-Ie aph (2”)-Ia* gene [17] which encodes high-level aminoglycoside resistance associated with transposons in *E. faecalis* (Tn*5281*), *Staphylococcus aureus* (Tn*4001*) and *Staphylococcus epidermidis* (Tn*4031*) [18].

### MLST of *E. faecalis*

All urine isolates identified as *E. faecalis* (n=27) were characterized by MLST to investigate if certain STs were associated with UTI. One of the three *E. faecalis* isolates collected from each patient was randomly selected and characterized by full MLST sequence analyses of all seven genes (*gdh, gyd, pstS, gki, aroE, xpt,* and *yqil*). To confirm pure culture in the urine samples, a second isolate from each of 16 (28.1%) samples was typed by sequence analyses of the *gki* and *yqil* genes. Since the DNA sequence of the *gki* and *yqil* genes were identical for the two isolates characterized from individual urine samples further sequencing were not done on isolates from the remaining urine samples. The PCR primers and conditions used were those described on the *E. faecalis*-MLST website ( http://efaecalis.mlst.net/). Amplicons were sequenced in both directions (Macrogen®). DNA sequences were assembled and a sequence type was assigned to each strain using CLC Main Workbench 5.2 software (CLC bio, Aarhus, Denmark) and compared to published alleles ( http://efaecalis.mlst.net/).

### PFGE

All urine isolates of ST 16 (n=12) were characterized by Pulsed-Field Gel Electrophoresis (PFGE) to determine their clonality and degree of similarity to four *E. faecalis* ST 16 strains isolated from Danish endocarditis patients [19]. DNA was prepared directly in a solid agarose plug (SeaKem Gold Agarose; Lonza, Basel, Switzerland) for restriction endonuclease digestion with the enzyme *Sma*I (BioLabs, New England, USA). The separations of fragments were done on CHEF-DR III (Bio-Rad, Richmond, CA, USA) at the following conditions: 6V/cm at 14°C for 20 h at a filed angel of 120°. The electrophoresis was carried out at switch times of 2.2 to 54.4 s. Following electrophoresis the gels were stained for 15 min in ethidium bromide (2 mg/ml water; Sigma-Aldrich Denmark A/S, Brøndby, DK), then destained in water for 15 min and visualized under UV light (Gel Doc, Bio-Rad) [20].

## Results

### Aetiology of UTI

Patients seen at the Hospital 103 with urine samples demonstrating pure cultures of ≥ 10^4^ CFU per ml were considered cases of UTI yielding a total of 49/276 (17.8%) positive urine samples. Among the 227 urine samples regarded as negative, coagulase-negative staphylococci appearing with small white colonies on the Flexicult agar plate were observed in 128 samples (56.4%), while 89 (39.2%) samples demonstrated < 10^4^ CFU per ml and/or mixed cultures, and 10 (4.4%) samples were sterile. Genus identification of bacterial colonies was based on their appearance on the chromogenic Flexicult agar according to the manufacturer’s instructions. Subsequently, species-specific PCR was used to identify *E. faecalis* and *E. faecium* and 16S rRNA sequence analysis was used to identify *Streptococcus* spp. and *Enterococcus* spp. which did not yield an amplicon in the *E. faecalis*- and *E. faecium* specific PCR. For *Streptococcus* spp. the results were confirmed by partial sequencing of *GroEL.* The bacterial aetiology could be established in 49 UTI cases (Table 1). 16S rRNA-based identification of four *S. gallolyticus subsp. pasteurianus* isolates was verified by sequencing of the *GroEL* gene and alignment with the sequence of the type strain [16] showing 100% similarity to the type strain. Thus, the sequencing of the *GroEL* gene thereby confirmed the 16S rRNA-based identification.

**Table 1:** Bacterial pathogens isolated from 49 patients with urinary tract infections (UTI) seen at a hospital in northern Vietnam

**Pathogen**	**No of isolates**
*E. faecalis*	27
*E. faecium*	2
*Enterococcus dispar*	1
*Enterococcus hirae*	2
*Enterococcus raffinosus*	1
*Enterococcus* spp.	1
*Streptococcus gallolyticus* subsp. *pasteurianus*	4
*Streptococcus peroris*	1
*E. coli*	6
*Staphylococcus* spp.	2
*Proteus* spp.	1
*P. aeruginosa*	1

### Characteristics of UTI patients

All 49 patients that showed ≥ 10^4^ CFU in pure culture per ml urine lived in the Ha Dong area in Hanoi. The mean age was 48 yrs (range was 11–86 yrs) and included 46 women and 3 men. On average, patients reported to have suffered from UTI symptoms for 348 days (range 3 days-10 yrs) at the time of their enrolment in the study. About one third (30.6%) of the patients had suffered from UTI previously. On average, patients who previously suffered from UTI had experienced 2.7 episodes during the last year (range 1–15 with the majority of patients experiencing between 1 to 5 episodes) of which 60.0% of the cases were diagnosed by a doctor. Treatment with antimicrobials before visiting the hospital was reported by 36.7% of UTI patients. However, only seven patients remembered which antimicrobial they took (chloramphenicol (2), ofloxacin (1), ampicillin (2), amoxicillin (1) and cephalexin (1)). Nine (18.4%) patients had used Chinese or Vietnamese herb medicine instead of, or in combination with antimicrobials. Specific symptoms were not observed for the different bacterial species associated with UTI.

### MLST and PFGE typing

All isolates identified as *E. faecalis* (n=27) by the species-specific PCR were characterized by MLST. A total of 12 sequence types (ST) was identified with ST 16 making up 44.4% of the isolates (Table 2).

**Table 2:** **Distribution of MLST sequence types, and allelic profiles among *E. faecalis *isolated from UTI patients in Vietnam**

**MLST**	**Number of isolates**	**Allelic profiles**						
**sequence type (ST)**		***gdh***	***gyd***	***pstS***	***gki***	***aroE***	***xpt***	***yqil***
ST 4	3	8	7	7	5	4	4	1
ST 16	12	5	1	1	3	7	7	6
ST 17	1	4	6	2	4	1	1	4
ST 93	2	25	2	15	9	23	18	26
ST 136	1	14	2	15	9	16	18	12
*ST 408	1	11	7	69	1	1	10	5
*ST 409	1	1	7	69	1	1	10	1
*ST 410	1	8	7	7	5	7	4	1
*ST 411	1	19	1	24	22	7	17	1
*ST 413	1	65	1	1	3	7	7	6
*ST 416	2	11	5	1	16	11	13	10
*ST 417	1	66	7	7	37	21	1	17

*New sequence types and allelic profiles.

**Figure 1:**
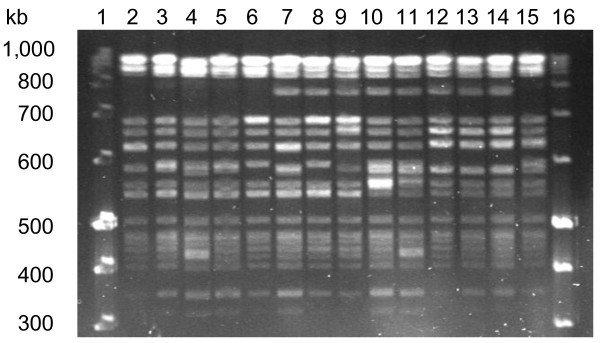
**Pulsed-field gel electrophoresis (PFGE) of *sma *I digested *E. faecalis *ST 16 strains.** Lanes 1 and 16 are molecular size markers. Lanes 2 to 11 show *E. faecalis* isolated from urinary tract infections in Vietnam and lanes 12 to 15 show *E. faecalis* isolated from Danish endocarditis patients. PFGE patterns of strains in lanes 2 to 6 and lane 15 are referred to as type A and lanes 7 to14 as type B.

The relatedness of PFGE types shown by the ST 16 isolates is shown in Figure 1. PFGE patterns of ten *E. faecalis* ST 16 isolated from UTI in Vietnam and four *E. faecalis* ST 16 isolated from patients with endocarditis in Denmark are shown as they represent the diversity seen for ST 16. All isolates shared 15 DNA fragments in their PFGE types. Five of the Vietnamese isolates and three of the Danish isolates (lanes 7 to 14) have an additional fragment of 750-kb size (Group B). Five Vietnamese isolates and one Danish isolate showed PFGE types without the 750-kb size fragment and are referred to as group A (lanes 2 to 6 and lane 15). When guidelines of Tenover *et al.*[21] are applied, all strains can be defined as closely related based on a maximum of three fragment difference.

### Antimicrobial susceptibility testing and *aac (6’)-Ie aph (2”)-Ia* gene

All *E. faecalis* isolates were susceptible to ampicillin, penicillin and vancomycin. Resistance against high-level gentamicin was common (48.1%). All of the high-level gentamicin-resistant isolates contained the *aac (6’)-Ie aph (2”)-Ia* gene (n=24) whereas the three isolates with a MIC <=16 μg per ml did not harbour the gene.

## Discussion

Our findings that *E. faecalis* was the most common pathogen associated with UTI are different to most previous studies where *E. coli* represents the main pathogen as e.g. in the international ECO.SENS study [3]. However, it should be mentioned that the ECO.SENS study as most other larger studies on UTI addressed only uncomplicated UTI cases. The patient characteristics found are in accordance with other studies with respect to age (mean age 48 yrs) and gender (94% women) [3]. However, the duration of symptoms and antimicrobial use patterns indicate recurrent, chronic and self-medicated UTIs. In our study, 30.6% of patients reported episodes of UTIs during the 12-month period before they enrolled in the study. Thus, the patients are likely to have suffered from recurrent UTIs and undertaken repeated self-medication, a practice which is described as widespread in Vietnam [10]. This situation may not be unique and can be expected in other countries with similar indiscriminate use of antimicrobials [22]. In addition, it appears as documented elsewhere [11] that the habit of some women to prevent and treat reproductive tract infections by vaginal douching with home prepared acidic solutions or commercial products as well as regular use of intra-vaginal antimicrobials may change the microflora in the genital tract and thus favor opportunistic infections. It is unknown to what extent such practices are associated with the atypical aetiology of UTI seen in our study. As antimicrobials are readily available at low costs for most Vietnamese, women suffering from acute UTI are likely to obtain antimicrobials for self-treatment at local pharmacies rather than signing up to take part in our study which will take their time and involve other people in their personal health problems. This may explain why mainly women suffering recurrent and complicated UTI (e.g. unknown underlying diseases) and showing atypical aetiology participated in our study.

About one third (37.6%) of the patients reported to have obtained antimicrobials from local pharmacies during a 12-month period before they were enrolled in the study; however, only seven patients were able to remember which type of antimicrobials they used. Further, patients were only asked if antimicrobials were used to treat UTI and they may have received additional antimicrobial treatments of other diseases. The first drug of choice used at the hospital to treat UTI was amoxicillin while the second drug of choice was cephalosporins which will select for *E. faecalis* due to its intrinsic resistance to cephalosporins [8]. Comparison of the prevalence of high-level gentamicin resistance reported from different geographic regions indicate that our finding of 48.1% resistant *E. faecalis* was higher than findings of 36% in a hospital in Denmark and 22% high-level gentamicin resistance in Greece [23,24], however, it should be noted that only a limited number of strains were included in our study. The gene *aac (6’)-Ie aph (2”)-Ia* was found in all strains showing high-level gentamicin resistance.

ST 16 made up 44.4% of the *E. faecalis* isolates recovered from UTI. In the MLST data-base ( http://efaecalis.mlst.net/), ST 16 is reported as the most frequent ST of *E. faecalis* recovered from clinical infections, faecal samples from hospitalized patients, healthy volunteers, and animals in Spain and the Netherlands [25]. The genotypes produced by PFGE confirms previous observations that *E. faecalis* ST 16 show little variation in PFGE patterns (1–3 fragment difference) which suggests that ST 16 is clonally distributed [25].

*S. gallolyticus* subsp. *pasteurianus,* formerly named *Streptococcus bovis* biovar II/2 [26,27] was the third most commonly isolated pathogen associated with four of the UTIs (Table 1). This species has previously been reported from cases of bacteraemia and endocarditis as well as urinary tract infections [26]. Murdoch *et al.*[28] reported *S. bovis* as an emerging pathogen associated with infective endocarditis and as the fourth most common cause of this disease in a multinational survey from 2000 to 2005. However, reports of UTI due to this organism are rare, and future investigations are needed to establish the role of *S. gallolyticus* subsp. *pasteurianus* in UTI.

## Conclusions

The aetiology of the UTIs seen among patients in Hanoi, Vietnam differed from acute uncomplicated UTI seen elsewhere as they were often recurrent, chronic and self-medicated. *E. faecalis* was the main pathogen associated with UTI in Vietnamese women and a possible selection associated with indiscriminate use of antimicrobials warrants further investigation. ST 16 was the dominating *E. faecalis* MLST type (44.4%) and their high genetic similarity as shown by PFGE to ST 16 isolates recovered from Danish endocarditis patients indicates that *E. faecalis* ST 16 is clonally distributed. Further studies are needed to establish the source(s) and role of *E. faecalis* ST 16 in UTI. The study was limited by a relative low number of UTI cases (49). Also, the aetiology of the UTI could have been strengthen by bacterial culture of two consecutive collected urine samples and using a definition of ≥ 10^5^ CFU per ml urine as a positive urine sample.

## Competing interests

The authors declare that they have no competing interests.

## Authors’ contributions

LLP participated in the study design, carried out laboratory work, analyzed the data and drafted and edited the manuscript. MB participated in the study design, analyzed data and edited the manuscript. NTS, NVT and HMA conceive participated in the study design and coordination and collected the specimens. AD participated in the study design, analyzed data and edited the manuscript. All authors have read and approved the manuscript.

## Pre-publication history

The pre-publication history for this paper can be accessed here:

http://www.biomedcentral.com/1471-2334/12/320/prepub
